# Два случая врожденного изолированного дефицита адренокортикотропного гормона вследствие патогенных вариантов в гене <i>TBX19</i>

**DOI:** 10.14341/probl13519

**Published:** 2025-07-22

**Authors:** Ю. Л. Скородок, А. В. Кожевникова, Е. В. Плотникова, И. Ю. Иоффе, А. Н. Тюльпаков

**Affiliations:** Санкт-Петербургский государственный педиатрический медицинский университет; Санкт-Петербургский государственный педиатрический медицинский университет; Санкт-Петербургский государственный педиатрический медицинский университет; Санкт-Петербургский государственный педиатрический медицинский университет; Медико-генетический научный центр им. акад. Н.П. Бочкова

**Keywords:** гипокортицизм, изолированный дефицит АКТГ, TBX19, гипогликемия, холестаз

## Abstract

Врожденный изолированный дефицит адренокортикотропного гормона (ВИДА) — орфанное аутосомно-рецессивное заболевание, обусловленное патогенными вариантами в гене ТВХ19 (1q24.2). В статье представлено описание двух клинических случаев с классической манифестацией ВИДА в неонатальном периоде, подтвержденного генетически, причем в одном из них выявлен впервые описанный вариант в гене TBX19. Диагноз установлен на 8-м и 22-м месяцах жизни, несмотря на появление клинически значимых симптомов в периоде новорожденности у обеих пациенток. Клинические проявления гипогликемии присутствовали у обеих пациенток: у пациентки №2 — с первых суток жизни (эпизод апноэ), у пациентки №1 — с 7 месяцев (судороги). У пациентки №1 основными проявлениями заболевания были холестатическая желтуха, гепатомегалия, признаки цитолиза гепатоцитов, нарушение белковосинтетической функции печени, что может свидетельствовать о развитии неинфекционного холестатического гепатита. Связь поражения печени с гипокортизолемией подтверждают улучшение и постепенная нормализация клинико-лабораторных изменений на фоне терапии гидрокортизоном. У пациентки №2 признаки холестаза отсутствовали. Лабораторно отмечались низкие уровни кортизола при сниженном или низконормальном — адренокортикотропного гормона (АКТГ), что подтверждает центральный гипокортицизм. При молекулярно-генетическом исследовании у обеих пациенток обнаружены патогенные варианты в гене ТВХ19 в гомозиготном состоянии: у пациентки №1 c.82C>T(p.Q28X), у пациентки №2 — c.469-1G>A, ранее не описанный.

## Abstract (en)

Congenital isolated ACTH deficiency (СIAD) is an orphan autosomal recessive disease caused by ТВХ19 (1q24.2) gene pathogenic variants. The article presents two cases with a classic manifestation of the СIAD in neonatal period which were confirmed genetically, one of cases revealed the first described TBX19 gene variant. Despite the appearance of significant symptoms in the newborn period in both patients diagnoses were established at the 8th and 22nd months of life. Clinical manifestations of hypoglycemia were present in both patients: from the 1st day of life (episode of apnoea) in patient № 2 and from 7 months (seizures) in patient № 1. Cholestatic jaundice, hepatomegaly, signs of hepatocyte cytolysis, impaired protein-synthetic liver function were the main manifestations of the disease in 1st patient. This may indicate non-infectious cholestatic hepatitis. Improvement and gradual normalization of clinical and laboratory symptoms during hydrocortisone therapy confirmed the association of liver damage with hypocortisolemia. 2nd patient had no signs of cholestasis. Low cortisol levels were observed with reduced or low-normal ACTH, which confirmed central hypocorticism. Genetic study in both patients revealed TBX19 gene pathogenic variants in a homozygous state: c.82C>T(p.Q28X) in patient №1, not previously described variant c.469-1G>A in patient №2.

## АКТУАЛЬНОСТЬ

Врожденный изолированный дефицит АКТГ (ВИДА) — орфанное аутосомно-рецессивное заболевание, обусловленное патогенными вариантами в гене ТВХ19 (MIM 604614), 1q24.2. Фактор транскрипции T-box (TPIT), кодируемый ТВХ19, необходим для терминальной дифференцировки клеток гипофиза, продуцирующих проопиомеланокортин, а также экспрессии гена POMC [1]. Заболевание с одинаковой частотой поражает мальчиков и девочек, в 40–42% отмечаются близкородственные браки [2][3]. В большинстве случаев ВИДА манифестирует в неонатальном периоде тяжелыми гипогликемиями, часто с приступами судорог и обструктивной желтухой [4]. Неспецифичность симптомов и низкая частота заболевания могут привести к поздней диагностике ВИДА [5][6], в связи с чем риск летального исхода в неонатальном периоде достигает 20–25% [3][7].

Представляем двух пациенток с ВИДА с классической манифестацией в неонатальном периоде (желтухой у первой, гипогликемией и апноэ у второй), подтвержденным генетически, причем у одной из них выявлен впервые описанный вариант в гене TBX19.

## КЛИНИЧЕСКИЙ СЛУЧАЙ

## Пациентка №1

Ребенок от второй физиологической беременности (1 беременность — медицинский аборт), родилась доношенной с массой тела 3750 г, длиной 52 см, малыми аномалиями развития: кранио-фациальные дисморфии и левосторонняя косолапость. Брак близкородственный: родители — двоюродные брат и сестра.

Неонатальный скрининг на врожденный гипотиреоз, врожденную гиперплазию коры надпочечников, галактоземию, фенилкетонурию, муковисцидоз — отрицательный.

В возрасте 1,5 месяца обследована в связи с затяжной желтухой и плохой прибавкой массы тела; отмечалась гипербилирубинемия (общий билирубин — 91 мкмоль/л; норма 8,5–20,5), гиперферментемия (АЛТ — 182,2 Ед/л; норма 5–30, АСТ — 144,5 Ед/л; норма 8–40, ГГТП — 80,8 Ед/л; норма 10–60), гипогликемия без клинических проявлений (глюкоза крови 2,2–2,7 ммоль/л; норма 3,3–5,5). При УЗИ выявлена гепатомегалия, при эластографии — фиброз печени легкой степени. Подтверждено наличие цитомегаловирусной инфекции (повышенный уровень Anti-CMV-IgG, положительная ПЦР на CMV), проведена противовирусная терапия без существенного эффекта.

Пациентка в 7 месяцев экстренно госпитализирована в связи с судорогами. Наблюдались нарушение сознания (сопор), мышечная гипотония, брадикардия (ЧСС 110 уд./мин.) и брадипноэ (ЧД 10 в 1 мин), артериальная гипотония (АД 65/40 мм рт.ст.), иктеричность кожных покровов, гепатоспленомегалия (печень +5,0 см, селезенка +2,0 см из-под реберной дуги), ахоличный стул, темная моча. Отмечалась высокорослость (длина тела — 73 см; SDS 2,03) при дефиците веса (масса тела — 7300 г, SDS ИМТ -2,38). В ходе обследования выявлены тяжелая гипогликемия (глюкоза крови — 0,6 ммоль/л; норма 3,3–5,5), гиперферментемия (АЛТ — 400 Ед/л; норма 5–30, АСТ — 180 Ед/л; норма 8–40), гипербилирубинемия (общий билирубин — 200 мкмоль/л; норма 8,5–20,5, непрямой — 142 мкмоль/л; норма 3,4–12), гипопротеинемия (общий белок — 53 г/л; норма 56–79), анемия (Hb — 97 г/л; норма 114–140, эритроциты — 3,69×10¹²/л; норма 4–5,3), гипокоагуляция (коалиновое время — 97 сек.; норма 50–70, тромбиновое время — 20 сек; норма — 15–18, ПТИ — 63,2%; норма — 78–142, МНО — 1,45; норма — 0,85–1,25, АПТВ — 74 сек.; норма 35–45). Исключено инфицирование Toxoplasma, H. simplex 1, 2, Mycoplasma hominis/pneumoniae, Chlamidia trahomatis/pneumoniae, HBV, HCV, VIH (методом ИФА), ЕВV, CMV, HНV (ПЦР), наследственные болезни обмена: аминоацидопатии, органические ацидемии, дефекты β-окисления жирных кислот (тандемная масс-спектрометрия).

При УЗИ подтверждена гепатоспленомегалия на фоне диффузных изменений паренхимы печени, холестаза.

В процессе дифференциальной диагностики сочетания персистирующей гипогликемии и неинфекционной желтухи выявлены сниженный уровень кортизола (15,9 нмоль/л; норма 138–635) при низком АКТГ (4,99 пг/мл; норма 8,17–46,32). Гиперинсулинизм, нарушение других тропных функций аденогипофиза и гипотиреоз исключены (табл. 1). Это позволило диагностировать ВИДА. Учитывая тяжесть состояния, назначена интенсивная терапия гидрокортизоном парентерально в дозе 75 мг/м²/сут. с последующим постепенным снижением под контролем клинических симптомов и гликемии до поддерживающей 10 мг/м²/сут. в таблетированном виде.

**Table:** Таблица 1. Уровни гормонов и глюкозы крови пациентки №1 в 7 месяцев Примечание. ТТГ — тиреотропный гормон, свТ4 — свободный Т4, СТГ — соматотропный гормон, ИФР-1 — инсулиноподобный фактор роста — 1.

Показатель	Результат	Норма
ТТГ, мМЕ/л	3,52	0,62–8,0
свТ4, пмоль/л	12,9	10–26
СТГ, нг/мл	3,5	1,3–9,1
ИФР-1, нг/мл	29,2	28–131
Инсулин, мкЕд/мл	0,2	2,3–26
С-пептид, нг/мл	0,1	0,36–3,6
Глюкоза, ммоль/л	0,6	3,3–5,5

При МРТ визуализированы арахноидальная киста левых отделов цистерны продолговатого мозга, диффузная корково-подкорковая атрофия больших полушарий головного мозга.

Для установления причины заболевания проведено секвенирование по Сэнгеру гена TBX19: у пациентки обнаружен ранее описанный [8] патогенный вариант (NM_005149.2.): c.82C>T(p.Q28X) в гомозиготном состоянии, у матери — в гетерозиготном состоянии (рис. 1 б, в).

**Figure:**
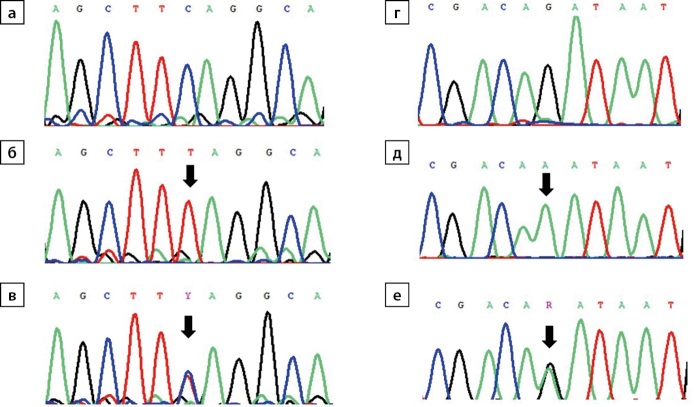
Рисунок 1. Хроматограммы с фрагментами сиквенса гена TBX19 (позиции с выявленным вариантом указаны стрелкой): а) фрагмент экзона 1 NM_005149.2:c.79_87 (последовательность дикого типа); б) гомозиготная транзиция C>T в положении NM_005149.2:c.82 (пациентка 1); в) гетерозиготная транзиция C>T в положении NM_005149.2:c.82 (мать пациентки 1); г) фрагмент стыка интрон 2/экзон 3 NM_005149.2:c.469-6_473 (последовательность дикого типа); д) гомозиготная транзиция G>A в положении NM_005149.2:c.469-1 (пациентка 2); е) гетерозиготная транзиция G>A в положении NM_005149.2:c.469-1 (отец пациентки 2).

На 5-й день терапии устранена гипогликемия (глюкоза — 4,1 ммоль/л), через 2 месяца разрешилась желтуха, снизилась активность ферментов печени (АЛТ=125 Ед/л, АСТ=93,3 Ед/л, ГГТП=65,1 МЕд/л), через 3 месяца сохранялись ускоренные темпы роста на фоне нормализации массы тела (длина тела — 79 см, SDS +2,57, масса тела — 9200 г, SDS по росту +0,16).

## Пациентка №2

Пациентка родилась от пятой беременности, протекавшей на фоне токсикоза и хронической фетоплацентарной недостаточности (старший брат 11 лет — здоров), вторых родов на 38-й неделе с массой тела 3340 г, длиной 52 см. Брак неродственный. В первые сутки отмечался эпизод апноэ, на вторые двукратно выявлена гипогликемия (глюкоза крови — 1,1 и 2,4 ммоль/л). В 2,5 месяца госпитализирована в неврологическое отделение в связи с повторным эпизодом апноэ и гипогликемическими судорогами (гликемия — 2,0 ммоль/л). Проведена КТ головного мозга, выявлены признаки смешанной гидроцефалии, субатрофии вещества головного мозга. Причина гипогликемии не установлена. Рекомендована вальпроевая кислота. В 3,5 месяца при плановом обследовании выявлены гипокортизолемия (кортизол — 27,6 нмоль/л; норма 138–635), умеренные гипертиреотропин- и гипотироксинемия (ТТГ — 8,9 мМЕ/л; норма 0,62–8,0, свТ4 — 9,05 пмоль/л; норма 11,5–20,4). В 4 месяца отмечалась высокорослость (длина тела — 68 см; SDS 3,1) при дефиците веса (масса тела — 6400 г, SDS ИМТ -2,6). В 7,5 месяцев госпитализирована в неврологическое отделение в связи с приступом тонико-клонических судорог на фоне поствакцинальной лихорадки. В ходе обследования неоднократно отмечалась гипогликемия (минимальная глюкоза крови — 0,59 ммоль/л). Диагностированы инфекционно-аллергический энцефалит (тяжелая форма), симптоматическая эпилепсия. В возрасте 1 года 9 месяцев повторно выявлена гипокортизолемия (кортизол — 24,4 нмоль/л) при низконормальном АКТГ 3,68 пг/мл (норма 0–30), диагностирован центральный гипокортицизм, начата терапия гидрокортизоном 15 мг/м²/сут. Подтвержден субклинический гипотиреоз (ТТГ — 16,3 мМЕ/л, свТ4 — 14,3 пмоль/л), назначен левотироксин — 12,5 мкг/сут.

В 2 года 1 месяц госпитализирована для контроля терапии и уточнения диагноза с жалобами на задержку психомоторного развития: начала ходить после года, не произносит слов. На фоне терапии гидрокортизоном в прежней дозе сохраняется высокорослость (рост — 98 см, SDS 3,8) и высокая скорость роста (19,1 см/год; SDS 3,9), масса тела нормализовалась (вес — 17 кг, SDS ИМТ 0,9); достигнута нормогликемия (глюкоза — 3,66–4,01 ммоль/л). Несмотря на нерегулярный прием левотироксина в минимальной дозе субклинический гипотиреоз не прогрессирует (ТТГ — 10,6 мМЕ/л; норма 0,64–5,76, свТ4 — 13,2 пмоль/л; норма 11,5–20,4). Проведено секвенирование по Сэнгеру гена TBX19, у пациентки обнаружен ранее не описанный в HGMD патогенный вариант (NM_005149.2.): c.469-1G>A в гомозиготном состоянии, у обоих родителей — в гетерозиготном состоянии (рис. 1 д, е).

## ОБСУЖДЕНИЕ

ВИДА вследствие патогенных вариантов в гене ТВХ19 крайне редкое заболевание, данные по заболеваемости и распространенности ограничены. В мировой литературе в основном освещаются единичные клинические случаи, которых насчитывается не более 80. Впервые доказательства связи ВИДА с дефектами в гене ТВХ19 были представлены в 2001 г. Lamolet с соавт., показавшими коэкспрессию генов Tpit и Pomc в клеточных линиях гипофиза мыши, и выявившими мутации в гене TPIT (TBX19) у 2 пациентов с вторичной надпочечниковой недостаточностью [9]. Abali Z.Y. и соавт. (2019 г.) обобщили данные о 66 ранее описанных пациентах, среди которых у большинства симптомы развивались в неонатальном периоде и заболевание диагностировалось до 2 лет [2][3][8].

Первые симптомы заболевания неспецифичны, что значительно затрудняет диагностику ВИДА [8]. Наиболее частыми симптомами считают гипогликемию (100%), в том числе симптоматическую (судороги, 53%), холестатическаую желтуху (62%), а также гипотермию или лихорадку, шок [3]. По данным ряда авторов, один из первых симптомов — приступ апноэ на 1–11-й дни жизни [1][10][11]. У некоторых пациентов развивается особая форма гепатита — неонатальный неинфекционный гепатит, связанный с гипокортизолемией; диагноз подтверждается при биопсии почти в каждом втором случае сочетания холестатической желтухи и гепатомегалии [8][11].

И в наших случаях диагноз установлен только на 8-м и 22-м месяцах жизни, несмотря на появление клинически значимых симптомов в периоде новорожденности у обеих пациенток, хотя гипогликемические судороги у пациентки №1 развились лишь к 7 месяцам, а у пациентки №2 отсутствовали признаки холестаза. Эпизод апноэ в первые сутки жизни у пациентки №2 следует расценить как результат гипогликемии. У пациентки №1 основными проявлениями заболевания были холестатическая желтуха (ахоличный стул, темная моча, гипербилирубинемия), признаки цитолиза гепатоцитов (гиперферментемия), нарушение белковосинтетической функции печени (гипоальбуминемия, гипокоагуляция), гепатомегалия. И хотя биопсию печени не проводили, данные изменения и отсутствие эффекта от противовирусной терапии могут свидетельствовать о развитии неинфекционного холестатического гепатита. Связь поражения печени с гипокортизолемией подтверждают улучшение и постепенная нормализация клинико-лабораторных изменений на фоне терапии гидрокортизоном. Учитывая вышесказанное, целесообразно при наличии гипогликемии ± холестаза у новорожденного или младенца исключить гипокортицизм.

Данные о росте пациентов с ВИДА вследствие патогенных вариантов в гене ТВХ19 противоречивы. Некоторые авторы описывают высокорослость на фоне нормального или сниженного ИФР-1 с нормализацией темпов роста на фоне заместительной терапии глюкокортикоидами [2][4][6]. Другие исследователи не выявили изменений антропометрических показателей у пациентов [12][13]. У обеих описываемых пациенток отмечалась высокорослость на фоне дефицита веса: умеренной степени к 7 месяцам (пациентка №1) и тяжелой в возрасте 4 месяцев (пациентка №2). Нормальный уровень СТГ и низконормальный — ИФР1 у пациентки №1 исключают связь высокорослости с нарушением в системе ГР-ИФР1. В отличие от литературных данных, высокие темпы роста на фоне терапии в течение 3–4 месяцев сохранялись при нормализации массы тела. Ввиду невозможности исследования уровня ИФР1-связывающего белка 5 подтвердить или опровергнуть гипотезу о связи высокорослости с отсутствием ингибирования этого белка при дефиците глюкокортикоидов, обусловленном ВИДА, не удалось [2].

Ряд авторов сообщали о различных дисморфологических признаках у пациентов с ВИДА, таких, как порок Арнольда-Киари 1-го типа и черепно-лицевые малые аномалии [3][4][6]. У пациентки №1 отмечались тригоноцефалия, скошенный затылок, узкие глазные щели, эпикант, антимонголоидный разрез глаз, широкая плоская переносица, микростомия, готическое небо, низко посаженные уши, левосторонняя косолапость. У пациентки №2 подобные аномалии не наблюдались. Установить связь фенотипа с патогенным вариантом гена ТВХ19 не представляется возможным. У обеих пациенток при визуализации выявлена диффузная атрофия/субатрофия вещества головного мозга, у пациентки №1 дополнительно — арахноидальная киста, у пациентки №2 — гидроцефалия. Подобных изменений ЦНС в литературе не описано, хотя в некоторых случаях отмечали умственную отсталость. Исключить связь субатрофии/атрофии вещества головного мозга у наших пациенток с тяжелыми гипогликемиями не представляется возможным.

Для ВИДА характерны резко сниженный уровень кортизола при сниженном [2][3][4][7][8][10][12] или нормальном [1][6] АКТГ. Следует отметить, что оценка уровня АКТГ зависит от чувствительности тест-систем. У наших пациенток отмечались аналогичные результаты: низкие уровни кортизола при сниженном или низконормальном АКТГ.

Среди пациентов с ВИДА описано несколько случаев идиопатического субклинического гипотиреоза [5][6][7][9]. В последующем уровни ТТГ нормализовались, в том числе спонтанно или на фоне лечения левотироксином в минимальных дозах. У пациентки №2 также была выявлена гипертиреотропинемия (в 3,5 месяца), сохраняющаяся в 2 года 1 месяц на фоне нерегулярного приема левотироксина 12,5 мкг/сут.

По данным на 2021 г., в HGMD (http://www.hgmd.cf.ac.uk) включены 29 патогенных вариантов в гене TBX19: ряд миссенс-мутаций, большие и малые делеции, четыре мутации в пределах сайта сплайсинга и новая синонимичная мутация NM_005149.3:c.288G>A (p.T96=) [1].

У пациентки №1 обнаружен патогенный вариант c.82C>T(p.Q28X) в гомозиготном состоянии, ранее выявленный Vallette-Kasic S и соавт., 2005 [8], что позволило подтвердить диагноз «ВИДА». Авторы описали сходные проявления у родных брата и сестры с данной мутацией: гипогликемию с судорогами, холестатическую желтуху, однако гепатит подтвержден не был и не отмечены какие-либо малые аномалии развития.

У пациентки №2 выявлен ранее не описанный вариант в гене ТВХ19 c.469-1G>A в гомозиготном состоянии. Хотя в настоящее время данный вариант отсутствует в референтных популяционных базах данных (https://gnomad.broadinstitute.org/), он может быть расценен как патогенный и каузальный, так как относится к влияющим на сплайсинг и присутствует в гомозиготном состоянии у пациента с классической клинико-лабораторной картиной ВИДА.

## ЗАКЛЮЧЕНИЕ

Представленные нами клинические случаи подчеркивают, что при наличии персистирующей гипогликемии, особенно в сочетании с холестазом, у новорожденного или младенца необходимо исключить гипокортицизм.

Выявление врожденного изолированного дефицита АКТГ (двукратно низкий уровень кортизола в сочетании с неповышенным АКТГ при отсутствии нарушения других функций аденогипофиза) требует молекулярно-генетического исследования.

В одном из представленных в настоящей публикации случаев выявлен ранее не описанный вариант в гене ТВХ19 (NM_005149.2.): c.469-1G>A.

## ДОПОЛНИТЕЛЬНАЯ ИНФОРМАЦИЯ

Источники финансирования. Молекулярно-генетическое исследование выполнено при частичном содействии Фонда поддержки и развития филантропии «КАФ».

Конфликт интересов. Авторы декларируют отсутствие явных и потенциальных конфликтов интересов, связанных с содержанием настоящей статьи.

Участие авторов. Все авторы одобрили финальную версию статьи перед публикацией, выразили согласие нести ответственность за все аспекты работы, подразумевающую надлежащее изучение и решение вопросов, связанных с точностью или добросовестностью любой части работы.

Согласие пациента. Пациенты добровольно подписали информированное согласие на публикацию персональной медицинской информации в обезличенной форме в журнале «Проблемы эндокринологии».

Благодарности. Выражаем благодарность Иванову Дмитрию Владимировичу, врачу педиатру, за своевременный диагностический поиск и оказание квалифицированной экстренной врачебной помощи пациентке №1.
